# Herpes simplex type 1 pneumonitis and acute respiratory distress syndrome in a patient with chronic lymphatic leukemia: a case report

**DOI:** 10.1186/s13256-017-1495-9

**Published:** 2017-11-23

**Authors:** Miriam Luginbuehl, Alexander Imhof, Alexander Klarer

**Affiliations:** 1 Department of Internal Medicine, University Hospital Zurich, Rämistrasse 100, 8091 Zurich, Switzerland; 2 Department of Internal Medicine, Division of Infectious Diseases, SRO AG Spital Langenthal, St. Urbanstrasse 67, 4901 Langenthal, Switzerland; 3 Interdisciplinary Intensive Care Unit, SRO AG Spital Langenthal, St. Urbanstrasse 67, 4901 Langenthal, Switzerland

**Keywords:** Herpes simplex virus, Pneumonia, Acute respiratory distress syndrome, Case report

## Abstract

**Background:**

Pulmonary pathogenicity of herpes simplex virus type 1 in patients in intensive care without classic immunosuppression as well as the necessity of antiviral treatment in the case of herpes simplex virus detection in respiratory specimens in these patients is controversial. We present a case of acute respiratory distress syndrome in a patient with stable chronic lymphatic leukemia not requiring treatment, in whom we diagnosed herpes simplex virus type 1 bronchopneumonitis based on herpes simplex virus type 1 detection in bronchoalveolar lavage fluid and clinical response to antiviral treatment.

**Case presentation:**

A 72-year-old white man presented with symptoms of lower respiratory tract infection. His medical history was significant for chronic lymphatic leukemia, which had been stable without treatment, arterial hypertension, multiple squamous cell carcinomas of the scalp, and alcohol overuse. Community-acquired pneumonia was suspected and appropriate broad-spectrum antibacterial treatment was initiated. Within a few hours, rapid respiratory deterioration led to cardiac arrest. He was successfully resuscitated, but developed acute respiratory distress syndrome. Furthermore, he remained febrile and inflammation markers remained elevated despite antibacterial treatment. Polymerase chain reaction from bronchoalveolar lavage fluid and viral culture from tracheobronchial secretions tested positive for herpes simplex virus type 1. We initiated antiviral treatment with acyclovir. Concomitantly we further escalated the antibacterial treatment, although no bacterial pathogen had been isolated at any point. Defervescence occurred rapidly and his C-reactive protein and leukocyte levels decreased. He was successfully weaned from mechanical ventilation, transferred to the ward, and eventually discharged to home.

**Conclusions:**

Herpes simplex virus should be considered a cause for lower respiratory tract infection in critically ill patients, especially in the setting of an underlying disease.

**Electronic supplementary material:**

The online version of this article (doi:10.1186/s13256-017-1495-9) contains supplementary material, which is available to authorized users.

## Background

Primary herpes simplex virus type 1 (HSV-1) infection occurs after viral inoculation at mucosal or skin surfaces. Symptoms range from severe, including fever, general malaise, and local vesicular rash, to frequently asymptomatic courses [1]. After primary infection, the virus may remain in a latent stage in nerve cell bodies. Reactivation can be triggered by physical or emotional stress, trauma to the site of primary infection, fever, and immunosuppression among other causes. Recurrence usually affects the site of primary infection, but may spread to adjacent areas via peripheral nerves or epithelial cells [2]. While rare in immunocompetent adults, HSV-1 infection of the lower respiratory tract (LRT) has been described in immunosuppressed and critically ill patients, where HSV-1 is frequently detected in LRT samples. According to a prospective study conducted in a Dutch University Hospital, the prevalence of HSV-1 in the upper respiratory tract of patients in an intensive care unit (ICU) is 22% compared with 3% in age and sex matched non-ICU patients [3].

Three different routes by which HSV-1 might infect the LRT have been proposed: contiguous spread via epithelial cells or aspiration from the oropharynx, hematogenous spreading, and neurogenic spreading along the vagus nerve after reactivation of latent infection in the vagal ganglion [4]. Reactivation of HSV-1 in the throat was shown to be an independent risk factor for HSV-1 detection in LRT samples of patients in ICU, which is compatible with spreading of the infection from oropharyngeal lesions to the LRT [3]. This hypothesis is further supported by a microsatellite analysis of HSV-1 isolates from the upper respiratory tract and LRT in patients who are mechanically ventilated. The microsatellite haplotypes were unique for every patient, but the same in upper respiratory tract and LRT samples of a specific patient [5].

Diagnosis of HSV infection of the LRT may be challenging, since specific clinical and radiologic features are lacking and the available laboratory tests have their limitations [4]. Available laboratory tests include direct antigen detection assays, virus culture, nucleic acid amplification tests, and serological assays, the latter being of little value in diagnosing acute HSV infections given the high frequency of seropositivity worldwide. Virus culture is more sensitive than direct antigen detection tests and is reported to be the gold standard diagnostic test [6]. The sensitivity of polymerase chain reaction (PCR) is even higher with virtually 100% specificity [6]. However, detection of HSV deoxyribonucleic acid by PCR or viral culture from respiratory specimens might not distinguish between simple viral shedding and invasive tissue infection, which can be demonstrated by histologic tissue examination or cytological examination for cytopathic effects of HSV infection. Instead, the use of quantitative real-time PCR has been proposed to differentiate between clinically relevant infection and simple viral shedding. A viral load of 10^4^ to 10^5^ gene equivalents per milliliter bronchoalveolar lavage (BAL) fluid was associated with a worse outcome [7, 8]. Whether HSV-1 causally worsens the outcome of serious illness or whether it is present as a consequence of serious illness remains unclear however. A real pathogenic role is supported by the results of a study in patients on prolonged mechanical ventilation (≥ 5 days) [9]. In this study, HSV bronchopneumonitis was defined as clinical deterioration, HSV detection in the LRT, plus HSV-specific nuclear inclusions. It was found to be common and associated with longer mechanical ventilation, longer ICU stay, and more episodes of bacterial ventilator-associated pneumonias without affecting in-hospital mortality [9]. Another study showed, however, no difference in respiratory impairment comparing survivors and non-survivors in whom HSV-1 was isolated from BAL fluid culture, while general disease severity (assessed by the Sequential Organ Failure Assessment score) independently predicted mortality [10]. The authors concluded that the presence of HSV-1 represents a marker of severe disease rather than contributing directly to morbidity and mortality [10]. These findings were supported by a prospective study, where HSV-1 loads in tracheal aspirates or BAL fluids, determined by real-time PCR, were only related to the Simplified Acute Physiology Score (SAPS) II [11].

Data regarding the need for treatment or pharmacologic prevention of HSV-1 in patients in ICU is scarce; large randomized controlled trials are lacking [12]. In a small randomized controlled trial published in 1987, prophylactic administration of acyclovir prevented the incidence of HSV effectively, but patient outcome did not improve [13]. In a retrospective analysis published in 2014, however, acyclovir treatment of patients in ICU with positive HSV-1 culture from BAL fluid was associated with reduced ICU mortality and in-hospital mortality [14]. Whether HSV-1 represents a relevant pathogen rather than a surrogate marker of severe disease and whether critically ill patients testing positive for HSV-1 need antiviral treatment remains to be investigated in interventional randomized controlled trials.

## Case presentation

We present a case of a 72-year-old white man presenting with general weakness, progressive dyspnea, and cough during the last days. His medical history was significant for chronic lymphatic leukemia (CLL), which had been stable without treatment, arterial hypertension, multiple squamous cell carcinomas of the scalp, and alcohol overuse. His regular medication consisted of atorvastatin, irbesartan, propranolol, and occasional use of chlorprothixene and diazepam. On physical examination, his temperature was 36.5 °C, blood pressure was 109/76 mmHg, pulse rate was 167 beats/minute and irregular, and respiration rate was 28 breaths/minute. A pulmonary examination revealed widespread rales. An electrocardiogram (ECG) showed newly diagnosed atrial fibrillation, which we judged as stress-related due to pulmonary infection. Blood tests showed increased C-reactive protein (CRP) levels (343 mg/L, normal < 6 mg/L), predominantly lymphocytic leukocytosis (68.9 × 10^9^/L) with a known baseline around 45 × 10^9^/L due to his CLL, elevated lactate level (3.6 mmol/L, normal < 1.6 mmol/L), hyperglycemia (10.3 mmol/L), mildly elevated liver enzymes (aspartate aminotransferase 105 U/l, normal < 51 U/l; alanine aminotransferase 110 U/l, normal 35 to 50 U/l), coagulopathy (international normalized ratio 1.3, normal 0.9 to 1.2), and mild macrocytic anemia (hemoglobin 11.6 g/dL, normal 14.0 to 17.5 g/dl). An arterial blood gas analysis showed hypoxemia and hypocapnia: partial pressure of oxygen (pO_2_) 66 mmHg, normal 70 to 90 mmHg; partial pressure of carbon dioxide (pCO_2_) 24.9 mmHg, normal 35 to 45 mmHg; bicarbonate 16.6 mmol/l, normal 22 to 24 mmol/l. An initial chest radiography was normal. His history of cough, new focal chest signs on clinical examination, and laboratory findings were consistent with community-acquired pneumonia. Because of his history of CLL and poor general condition, we started a broad-spectrum intravenously administered antibiotic regimen with piperacillin/tazobactam. In addition, anticoagulation with heparin (bolus and infusion) was started for atrial fibrillation.

**Fig. 1:**
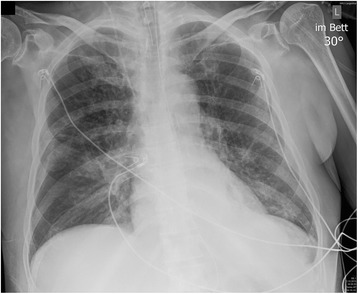
Bilateral pulmonary infiltrates. Chest radiography on the fifth day of hospitalization showing bilateral acinar infiltrates consistent with acute respiratory distress syndrome

Within a few hours, he developed rapidly progressive respiratory failure leading to cardiac arrest. As the cause of this respiratory failure, we identified pulmonary infection. Notably, there were no clinical signs of anaphylaxis or any known drug allergies from his history. Chest radiography excluded pneumothorax. Acute pulmonary embolism seemed unlikely as therapeutic anticoagulation had already been started for atrial fibrillation. After cardiopulmonary resuscitation and intubation, he was stabilized in our ICU. Oxygenation remained impaired and consequent chest radiographies demonstrated progressive bilateral infiltrates (Fig. 1). Echocardiography showed normal left and right ventricular function, thus we diagnosed mild acute respiratory distress syndrome. Weaning from mechanical ventilation was prolonged, so a dilatation tracheostomy was performed. After initially decreasing CRP levels and leukocyte counts, he remained febrile and inflammation markers remained elevated even after addition of clarithromycin to the antibiotic regimen. All microbiologic samples initially taken, including blood cultures, tracheobronchial secretions culture and Gram stain, and *Legionella* and pneumococcal urinary antigen, were negative. A BAL was performed and HSV-1 PCR from BAL fluid tested positive. Due to a too small sample volume, we used tracheobronchial secretions for testing HSV-1 in viral culture, which also tested positive. The BAL fluid culture showed growth of *Candida albicans*. All other microbiologic examinations were negative, including serology for *Mycoplasma*, *Mycobacterium tuberculosis* PCR, microscopy, and culture as well as *Pneumocystis jirovecii* PCR and microscopy, galactomannan enzyme immunoassay (EIA), and PCR for adenovirus, influenza A and B, and other respiratory viruses from BAL fluid. He was started on intravenously administered acyclovir 10 mg/kg three times daily. Concomitantly, the antibacterial treatment was escalated to imipenem/cilastatin for suspected bacterial superinfection given a mildly elevated procalcitonin (1.54 mcg/L, normal < 0.10 mcg/L). Defervescence occurred rapidly, his CRP level decreased to 25 mg/L, leukocyte counts returned to the former baseline of 40 to 50 × 10^9^/L, and the infiltrates on consequent chest radiographies improved markedly. Antiviral treatment was continued for 14 days; antibacterial treatment was given for 10 days. After successful weaning from mechanical ventilation he was transferred to the ward and discharged to home after 29 days (Additional file 1: Figure S1).

## Discussion

Clinicians are confronted with controversial data regarding pulmonary pathogenicity and the necessity of treatment of HSV-1 in patients in ICU. Which patients should be treated is still an individual decision based on the clinical context. Our patient showed clinical deterioration, respiratory impairment, and persistently elevated inflammation markers despite broad-spectrum antibiotic treatment. We assumed underlying immunodeficiency due to his CLL, which was supported by evidence of reduced total immunoglobulin G levels (3.6 g/L, normal 7.0 to 16.0 g/L). No bacterial pathogen could be isolated at any point. Given these circumstances, we interpreted HSV-1 detection as evidence of clinically relevant infection warranting antiviral therapy. Underlying immunodeficiency led to exclusion from many studies of HSV bronchopneumonitis in patients in ICU. Data for this special subset of patients in intensive care are thus scarce to date.

## Conclusion

We highlight with this case that atypical and opportunistic infections, such as herpes viruses, should be liberally considered in patients with underlying disease.

## Additional file

Additional file 1: Timeline. (PDF 29 kb)

## Abbreviations

BAL: Bronchoalveolar lavage
CLL: Chronic lymphatic leukemia
CRP: C-reactive protein
ECG: Electrocardiogram
EIA: Enzyme immunoassay
HSV-1: Herpes simplex virus type 1
ICU: Intensive care unit
LRT: Lower respiratory tract
pCO_2_: Partial pressure of carbon dioxide
PCR: Polymerase chain reaction
pO_2_: Partial pressure of oxygen
SAPS: Simplified Acute Physiology Score
