# Supplementary material for: Herpes simplex type 1 pneumonitis and acute respiratory distress syndrome in a patient with chronic lymphatic leukemia: a case report
Source: J Med Case Rep. 2017 Nov 23;11:329. doi: 10.1186/s13256-017-1495-9 (PMC5700675; doi:10.1186/s13256-017-1495-9)
Supplement: Additional file 1: — Timeline. (PDF 29 kb) [file 13256_2017_1495_MOESM1_ESM.pdf]

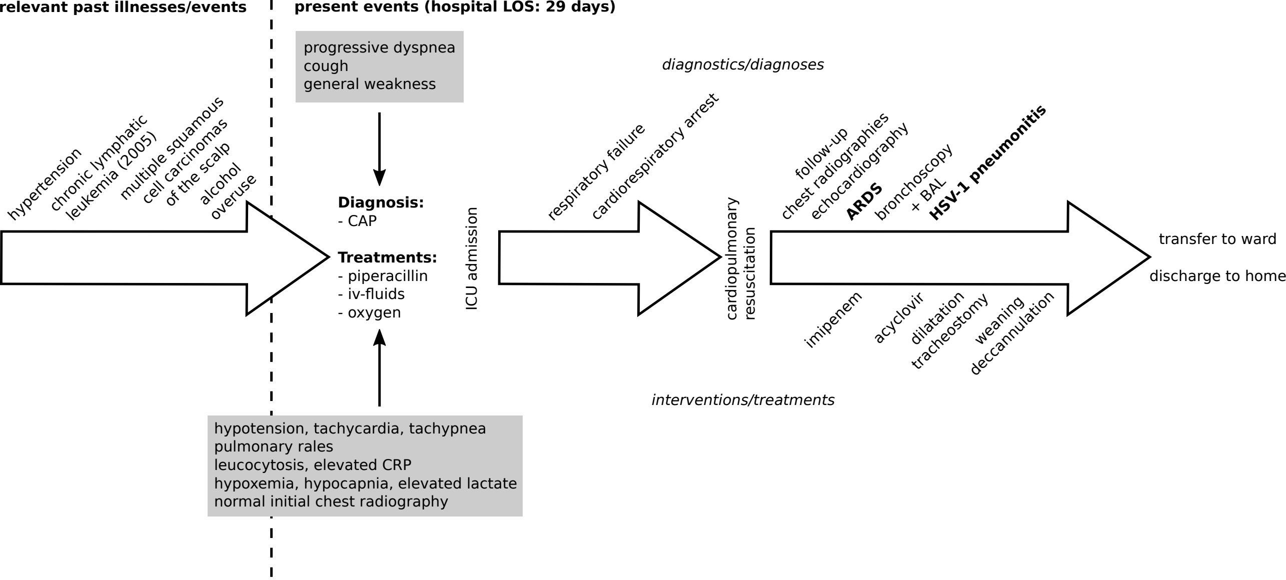

Timeline. Abbreviations: LOS = length of stay, CRP = C-reactive protein, CAP = community-acquired pneumonia, ARDS = acute respiratory distresssyndrome, HSV-1 = Herpes simplex type 1
